# Analysis of Prevalence, Socioeconomic and Disease Trends of Non-Melanoma Skin Cancer in New Zealand from 2008 to 2022

**DOI:** 10.1007/s44197-024-00250-4

**Published:** 2024-06-06

**Authors:** Sharad Paul, Yipan Chen, Mahsa Mohaghegh

**Affiliations:** 1 Auckland University of Technology, 55 Wellesley Street East, Auckland, 1010 New Zealand; 2 Skin Surgery Clinic, 271A Blockhouse Bay Road, Auckland, 0600 New Zealand

**Keywords:** Skin cancer, Basal cell carcinoma, Squamous cell carcinoma, Prevalence, New Zealand, Non-melanoma skin cancer

## Abstract

**Background:**

Skin cancer shows geographic and ethnic variation. New Zealand—with a predominantly fair-skinned populations, high UV indices and outdoor lifestyles—has high rates of skin cancer. However, population prevalence data is lacking. This study aimed to determine the demographics and socioeconomic disease trends of non-melanoma skin cancer prevalence in New Zealand from a large targeted-screening study.

**Methods:**

A targeted screening programme was conducted among 32,839 individuals, Fitzpatrick Skin Types I to IV in Auckland, New Zealand during the 2008–2022 period. This data was analyzed retrospectively. Linear regression models were used to assess statistical trends of skin cancer prevalence over time, along with associated factors that included demographics, disease trends and overall prevalence.

**Results:**

A total of 32,839 individuals were screened and 11,625 skin cancers were detected. 16,784 individuals were females who had 4,378 skin cancers. 16,055 individuals were males who had 5,777 skin cancers. 54 males and 65 females had multiple skin cancers. The article presents detailed descriptions of tumour types and subtypes detected, age groups, demographic and socioeconomic information. regarding the non-melanoma skin cancers detected.

**Conclusion:**

Overall men have more non-melanoma skin cancer (NMSC) than females; however females develop more BCC on the lips. BCC is three times more common in the 31-50 age group, whereas SCC are significantly more prevalent after age 80. Prevalence of BCC has not changed over the 15-year timeframe of the study but SCC has increased. Older ages and higher incomes are associated with higher rates of NMSC in New Zealand.

## Introduction

Non-melanoma skin cancers (NMSC) are the most common tumours diagnosed globally, with an annual increase in prevalence of NMSC due to ageing populations [1]. New Zealand has one of the highest prevalence of NMSC in the world [2] and the incidence of NMSC in the Auckland region of New Zealand was estimated per 1906.5 per 100,000 [3]. However, true prevalence data is lacking as this is not routinely collected by cancer registries. While screening of all populations for skin cancer is not recommended [4], the usefulness of targeted screening of high-risk populations—typically with fair skin and therefore with a higher propensity for sun damage—has well been established for skin cancer [5].

Among skin cancers, keratinocyte carcinomas, i.e., basal cell carcinoma (BCC) and squamous cell carcinoma (SCC), account for most lesions, but as most cancer registries do not record these, both incidence and prevalence data have been lacking internationally [6]. In Australia, a report to Queensland Health in 2012 estimated the prevalence of BCC and SCC to be 5% and 2%, respectively (in men), and 4% and 1%, respectively (in women), but this was based on a telephone call and self-reporting, with no clinical assessment of diagnosis or pathology correlation [7]. The prevalence of keratinocyte skin cancers globally has significantly increased over the last 20 years, with reports estimating rises of 35% for BCC and 133% for SCC [8]. Another report noted that NMSC increased internationally between 1990 and 2017 by 310% for squamous cell carcinomas (SCCs) and 77% for basal cell carcinomas (BCCs), with further increases forecast [9]. A Victorian government report in Australia estimated that BCC and SCC accounted for 8.1% of all health system spending on cancer in Australia [10]. New Zealand has had a problem with capturing data because NMSC are not recorded by cancer registries, and majority of skin cancers are treated in primary care, and therefore the size of the skin cancer population burden remained unknown [11]. To answer this question, the Skin Surgery Clinic, a skin cancer services provider undertook a mostly cost-free targeted screening population study to investigate and shed light on the prevalence of NMSC in higher-risk, predominantly Caucasian (Fitzpatrick Skin Types 1–4) population in New Zealand over an extended timeframe. This screen included a full body clinical examination by a clinician that was correlated with pathology reports from biopsies performed when suspicious lesions were identified. tumour types, anatomical locations, demographic and socioeconomic patterns are recorded here.

## Materials and methods

### Subjects and Study Design

This cross-sectional study was conducted among 32839 people that underwent skin cancer screening checks during 2008 to 2022.

The inclusion criteria were as follows:

1. Fitzpatrick Type 1 to Type 4 skin.
2. Voluntarily amenable to undergo full body skin examination.
3. Able and willing to provide informed consent for a biopsy of any suspicious lesions identified.

The exclusion criteria were as follows:

1. A known skin cancer i.e., someone referred to the Clinic or other clinics with a known skin cancer lesion.
2. Age under 18, or over than 100.
3. Unable to provide informed consent.
4. Screening performed outside 2008–2022.
5. Faulty registrations i.e., demographic data missing.

### Data Collection

This retrospective study is based on data collected at the Skin Surgery Clinic, a private skin cancer clinic, which conducted a targeted screening programme of people with fair skin—predominantly cost-free to remove any socioeconomic barriers to participation. The participants were mostly from the greater Auckland region with a catchment that represents slightly under a third of New Zealand’s overall population of 5.2 million. Compared to any other such study, we believe this large screening project offers a more comprehensive understanding of population prevalence of skin cancer because in addition to tumour sub-types and anatomical locations, demographic and socioeconomic data were also recorded.

### Screening Protocols and Procedures

Recruitment: Participants were recruited initially via advertisements in the suburban newspapers and radio. While as the study became known volunteers did attend directly, the exclusion criteria still applied.

Questionnaire: Basic information from the screening subjects included age, gender, ethnicity, income decile (when available), occupation, pfamily history, and past history of skin cancer.

Full skin examination: All participants underwent a full skin examination (including scalp and feet), and any lesions of note were examined with a dermatoscope. Fitzpatrick skin type was recorded.

Histological examination: Any suspicious lesions were biopsied (either shave, punch, incisional or excisional biopsy depending on which was the most appropriate). Pathology specimens were analysed by histopathologists at the only lab that services the greater Auckland region. All NMSC recorded were from confirmed biopsies.

### Data Quality Control

**Fig. 1:**
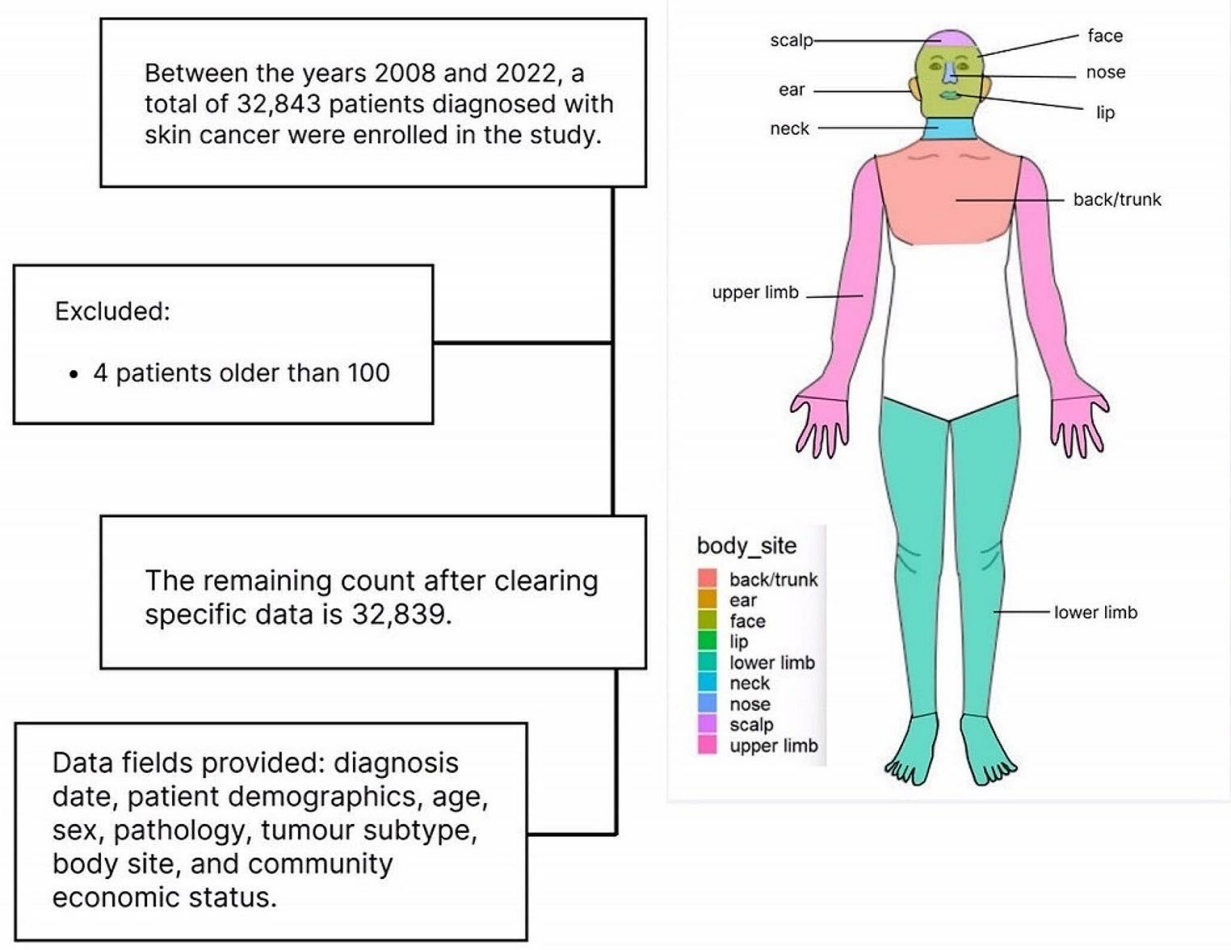
Flow diagram of data collection and analysis

The flow chart of the data collection is depicted in Fig. 1. After the study data was collated, it was confirmed as complete and correct by an independent quality controller before it was passed onto investigators undertaking the statistical study of this database to ensure data quality.

The dataset used in this study contains information as presented in Table 1. Individual-level data was collected using the following variables: diagnosis date, name, age, gender, quintile (representing socioeconomic status), disease site (specific body sites affected by skin cancer), and types of skin cancer (including tumour subtypes). The diagnosis code recorded was based on the pathology report.

Age groups at diagnosis were categorized into specific age bands, utilizing 10-year age ranges. These groups included individuals aged < 20 years, 21–30 years, 31–40 years, and so on. This ensured adequate data within each age group to ensure statistically significance. Patients younger than 18, and older than 100 years of age were excluded from the analysis to avoid any skewing of data.

The study focused on individuals predominantly in Auckland belonging to Fitzpatrick skin types 1–4. This research predominantly consisted of individuals of European descent including fair-skinned Māori. At the 2018 census, 70% of New Zealand’s population were of fair-skinned European descent (often referred to by the Māori term, Pākehā).

Socioeconomic status was recorded in 5 quintiles bands. New Zealand ranks the economic levels of different regions, from 1 (least deprived) to 6,181 (most deprived) and these are usually divided into five quintiles. Quintile 1 indicates 20% least socioeconomically deprived regions in New Zealand, while quintile 5 represents the 20% most socioeconomically deprived areas [12].

### Statistical Methods

Data collation and analysis have been undertaken using various methods including R, Python, and Power Bi software. Differences between groups were studied using the Chi-squared test, and a P value of < 0.05 was considered statistically significant. Furthermore, linear regression analysis was utilized to assess statistical trends to determine if the prevalence of BCC and SCC varied over time.

Descriptive statistical analyses were applied to explain the demographic and socioeconomic characteristics of the skin cancer screening programme from 2008 to 2022. Categorical variables were displayed as numbers (%). We describe the year-to-year changes, overall NMSC detection rates and include the median of the annual detection rates as a continuous variable for trend analysis. We also detail the diagnosis of skin cancer by tumour subtype and anatomical location for each year based on the screening population.

#### Skin Cancer Subtypes

While actinic keratoses (AK) are not precursor of BCC, the presence of AK on someone’s skin indicates actinic damage and may be involved in BCC progression [13]. BCC subtypes varied from the relatively low-risk superficial BCC and nodular BCC to the more aggressive infiltrating, morphoeic and sclerosing BCC, with micronodular BCC known to have a higher risk of recurrence [14] [15].. SCC subtypes tend to occur more sequentially, starting with SCC in-situ and as differentiation lessens, prognosis worsens i.e., well-differentiated SCC are less likely to spread than moderately differentiated SCC, which in turn has a better prognosis than poorly differentiated SCC [16].

### Ethics

All participants had given informed consent to participate in the screening programme. As this study only retrospectively examined, already deidentified and depersonalised data derived from participant records,the institutional committee of the Auckland University of Technology concluded that formal ethics approval was not applicable.

## Results

The flow diagram of the Study is illustrated in Fig. 1. A total of 32,839 individuals were screened and 11,625 skin cancers were detected. 16,784 individuals were females who had 4,378 skin cancers. 16,055 individuals were males who had 5,777 skin cancers. 54 males and 65 females had multiple skin cancers. The article presents detailed descriptions of tumour types and subtypes detected, age groups, demographic and socioeconomic information. regarding the non-melanoma skin cancers detected.

The prevalence of non-melanoma skin cancers (within the screened population) increased with age, with the prevalence of BCC increasing significantly from 0.25% at 0–20 years of age, to 21.76% at 71–80 years of age, and then a slower increase to 24.58% at 91–100 years of age. Prevalence of SCC showed a different pattern. SCC linearly increased from 0% at 0–20 years of age to 36.99% at 91–100 years of age.

Demographic factors vary for different skin cancers (Table 1). The prevalence of skin cancer within the study population increased progressively with age, especially after age 41. The prevalence of skin cancer was higher in males (39.3%) than in females (28.1%).

**Table 1:** NMSC Prevalence data expressed as a percentage of the study population

Total		Count	BCC ICD-10 code C44. 91		SCC ICD-10 code C44. 92	
Age group		N	N	%	N	%
	0–20	397	1	0.25	0	0
	21–30	1048	16	1.53	4	0.38
	31–40	2359	147	6.23	36	1.53
	41–50	3956	498	12.59	148	3.74
	51–60	6215	1019	16.40	540	8.69
	61–70	7912	1525	19.27	1131	14.29
	71–80	6995	1522	21.76	1464	20.93
	81–90	3538	830	23.46	1023	28.91
	91–100	419	103	24.58	155	36.99
Gender						
	Male	16,055	3355	20.9	2422	15.09
	Female	16,784	2300	13.7	2078	12.38
Year						
	2008	2085	291	13.96	147	7.05
	2009	4723	744	15.75	443	9.38
	2010	7556	1225	16.21	841	11.13
	2011	10,573	1746	16.51	1239	11.72
	2012	13,800	2302	16.68	1741	12.62
	2013	16,848	2826	16.77	2177	12.92
	2014	19,820	3385	17.08	2617	13.20
	2015	21,890	3807	17.39	2923	13.35
	2016	23,696	4184	17.66	3214	13.56
	2017	25,372	4495	17.72	3488	13.75
	2018	27,293	4846	17.76	3797	13.91
	2019	28,671	5069	17.68	4040	14.09
	2020	30,785	5483	17.81	4342	14.10
	2021	32,328	5780	17.88	4604	14.24
	2022	32,839	5884	17.92	4688	14.28
Body site						
	back/trunk	9783	1955	33.32	533	11.40
	ear	873	213	3.63	182	3.89
	eye	32	9	0.15	3	0.06
	lip	471	69	1.18	64	1.37
	face	9095	1775	30.25	1482	31.69
	lower limb	4844	717	12.22	1005	21.49
	neck	165	50	0.85	20	0.43
	nose	1141	358	6.10	133	2.84
	scalp	1142	106	1.81	247	5.28
	upper limb	4723	615	10.48	1007	21.54
Quintile						
	1	5661	1070	3.26	826	2.52
	2	5025	937	2.85	819	2.49
	3	4285	850	2.59	697	2.12
	4	2892	593	1.81	436	1.33
	5	1012	213	0.65	172	0.52

*Note* Data are shown as number (%) or number (N)

**Fig. 2:**
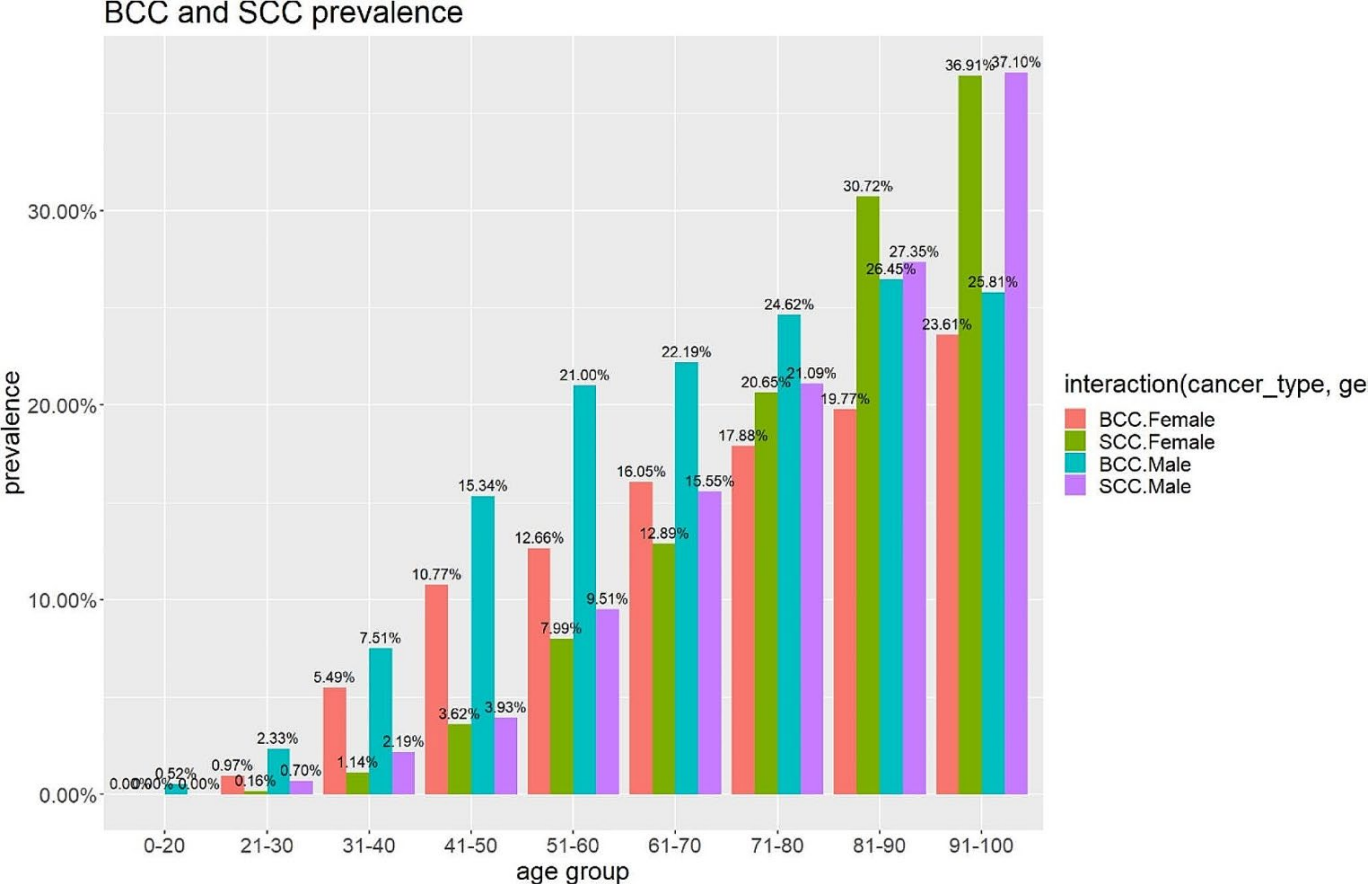
Prevalence of BCC and SCC between genders at different age groups

Figure 2 illustrates the findings by population and timeline. The data showed that SCC prevalence increased more than BCC over the years. The rise was most notable between 2008 and 2012, with a jump from 7 to 12.9% of the study population, followed by a gradual increase to its peak of 14.3% in 2022. There was no change in recruitment methodology or statistically relevant change in numbers to account for this.

For the whole cohort, Fig. 2 illustrates the variation of NMSC across different age groups. Our analysis indicates that the prevalence of NMSC progressively increases with age. We observed a significant rise in the prevalence of BCC between ages 41 and 80 (18.7[95% CI 18.6–18.8]) followed by a more gradual increase. Prevalence of SCC showed a positive correlation with age and increased significantly after age 40, surpassing the prevalence of BCC after age 70 (24.9[95% CI 24.8–25.1]).

While prevalence of NMSC rises with age, there is a significantly higher prevalence of BCC in males than females but also a reversal in prevalence between genders as females surpass males in SCC prevalence after age 80.

**Fig. 3:**
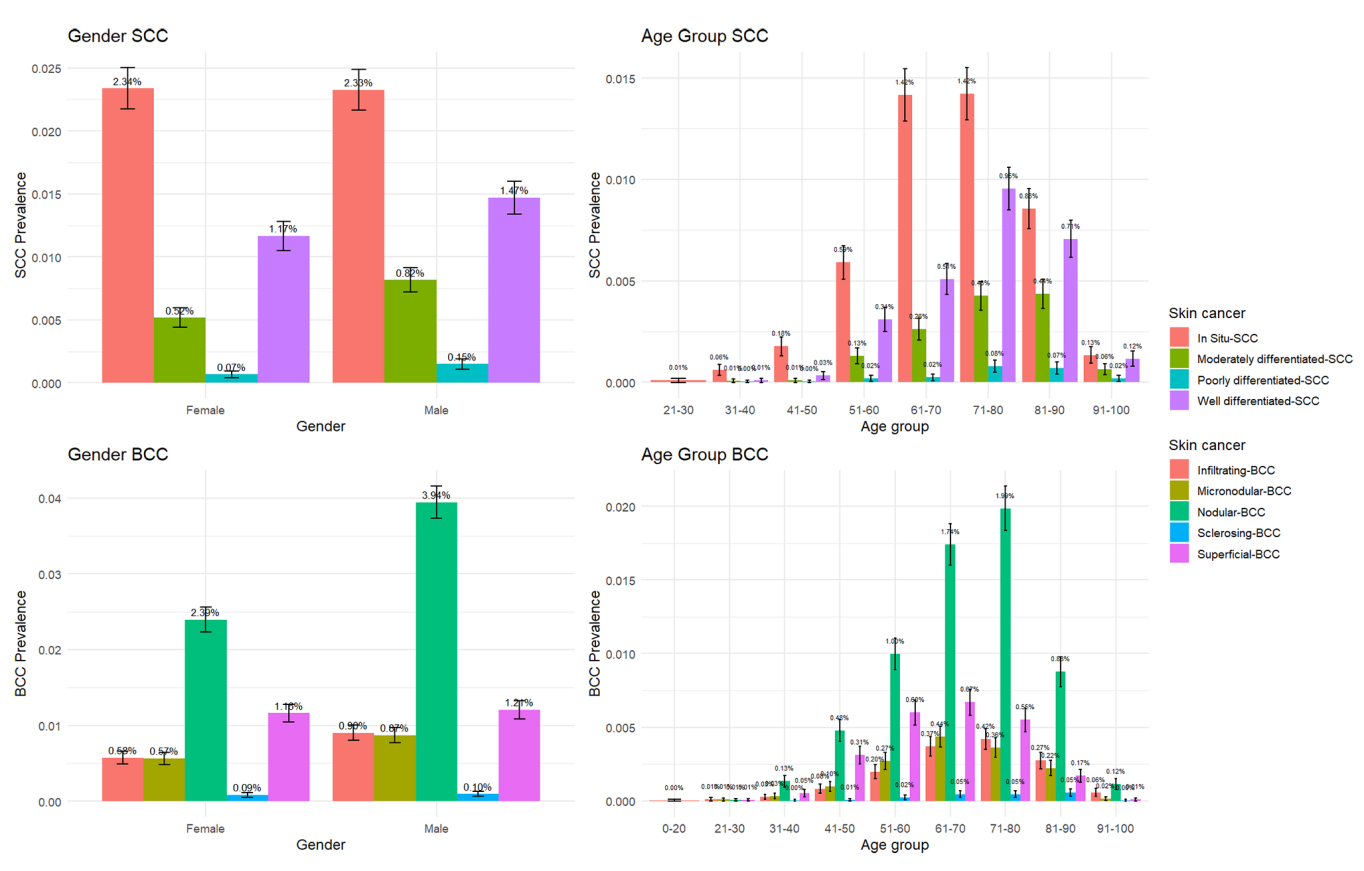
Prevalence of subtypes of BCC and SCC in New Zealand by age group and gender (colour- code representation of body anatomical sites from Fig. 1)

Among the BCC subtypes by age (Fig. 3), nodular BCC had the highest prevalence in the 71–80 age group (1.98 [95% CI 1.83–2.14]), and superficial BCC had the highest prevalence in the 61–70 age group (0.67 [95% CI 0.58–0.76]). Among the BCC subtypes by gender the prevalence of nodular BCC was significantly higher in males (3.94 [95% CI 3.73–4.15]) than in females (2.39 [95% CI 2.23–2.56]). Among the SCC subtypes, SCC in-situ had the highest prevalence in the age group of 71–80 years (1.42 [95% CI 1.29–1.55]). By gender, the prevalence of SCC in-situ in males (2.33 [95% CI 2.16–2.49]) was similar to the prevalence of SCC in-situ (2.34 [95% CI 2.18–2.5]) in females. The highest prevalence of the different subtypes of non-melanoma skin cancer were in the 61–70 and 71–80 age groups, and for the BCC subtypes the prevalence was slightly higher in males than in females, while for the SCC subtypes the prevalence was similar among genders.

**Table 2:** Tumour numbers by subtype of non-melanoma skin cancer (NMSC) in this study

Tumour Subtype	Number
BCC (unspecified)	2029
SCC in-situ	1487
Infiltrating BCC	486
Micronodular BCC	472
Moderately differentiated SCC	68
Nodular BCC	1908
Poorly differentiated SCC	68
SCC (unspecified)	1520
Sclerosing BCC	48
Superficial BCC	740
Well differentiated SCC	795

**Fig. 4:**
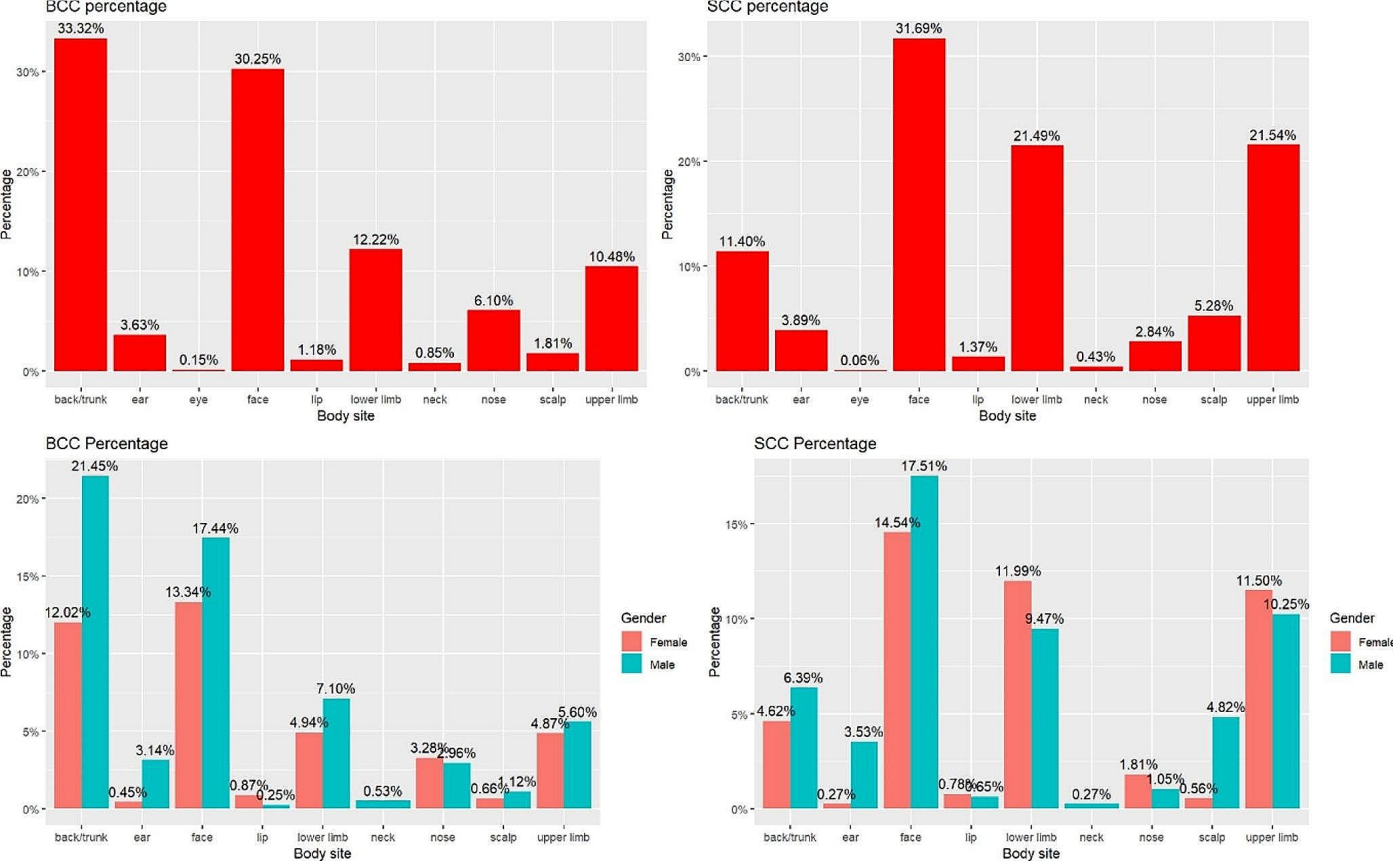
Prevalence and gender differences in body site and the type of NMSC in the study population

The occurrence of NMSC on different body sites was analysed and displayed in Figs. 1 and 4. The results show that NMSC in the study population was mainly concentrated on the face (27.7[95% CI 27.2–28.2]), back (29.8[95% CI 29.3–30.3]), and limbs (29.1[95% CI 28.6–29.6]), accounting for over 70% of cases,with lesser occurrences on the nose (3.5[95% CI 3.3–3.7]), ears (2.7[95% CI 2.5–2.8]), neck (0.5[95% CI 0.4–0.6]), and scalp (3.5[95% CI 3.3–3.7]). Statistical analyses of anatomical locations indicated that the probability of SCC is significantly higher on the face (31.69%), lower limbs (21.49%), and upper limbs (21.54%) compared to the back region (11.40%) (*p* < 0.05). However, the back (33.32%) and face (30.25%) have a significantly higher probability of BCC occurring compared to the lower limbs (12.22%) and upper limbs (10.48%) (*p* < 0.05). Further investigation revealed gender-specific variations, with a higher prevalence of BCC in females more than males on the lip. Additionally, females have a higher prevalence of SCC on the nose and limbs than males, although NMSC prevalence is overall higher in males.

**Table 3:** Age group and gender characteristics of non-melanoma skin cancer carcinomas in New Zealand by subtype

	Infiltrating- BCC		Micronodular- BCC		Nodular- BCC		Superficial- BCC		Sclerosing- BCC	
Age group	N	%	N	%	N	%	N	%	N	%
0–20	1	0	0	0	0	0	0	0	0	0
21–30	4	0.01	3	0.01	2	0.01	2	0.01	0	0
31–40	9	0.03	11	0.03	44	0.13	17	0.05	1	0
41–50	27	0.08	32	0.10	157	0.48	102	0.31	2	0.01
51–60	65	0.20	89	0.27	328	1.00	197	0.60	9	0.02
61–70	122	0.37	143	0.44	572	1.74	220	0.67	15	0.05
71–80	138	0.42	119	0.36	652	1.99	181	0.55	15	0.05
81–90	90	0.27	73	0.22	288	0.88	56	0.17	18	0.05
91–100	19	0.06	5	0.02	38	0.12	3	0.01	1	0
Gender	N	%	N	%	N	%	N	%	N	%
Male	297	0.90	286	0.87	1295	3.94	396	1.21	32	0.10
Female	189	0.58	186	0.57	786	2.39	382	1.16	28	0.09
			SCC in-situ		Moderately diff. SCC		Poorlydiff. SCC		Well diff. SCC	
Age group	N	%	N	%	N	%	N	%	N	%
0–20			0	0	0	0	0	0	0	0
21–30			3	0.01	0	0	0	0	0	0
31–40			20	0.06	2	0.01	1	0	3	0.01
41–50			58	0.13	3	0.01	1	0	11	0.03
51–60			194	0.59	43	0.13	6	0.02	102	0.31
61–70			465	1.42	86	0.26	8	0.02	167	0.51
71–80			467	1.42	140	0.43	26	0.08	313	0.95
81–90			281	0.86	143	0.44	23	0.07	232	0.71
91–100			44	0.13	21	0.06	6	0.02	38	0.12
Gender	N	%	N	%	N	%	N	%	N	%
Male			764	2.33	268	0.82	49	0.15	483	1.47
Female			768	2.34	170	0.52	22	0.07	383	1.07

**Fig. 5:**
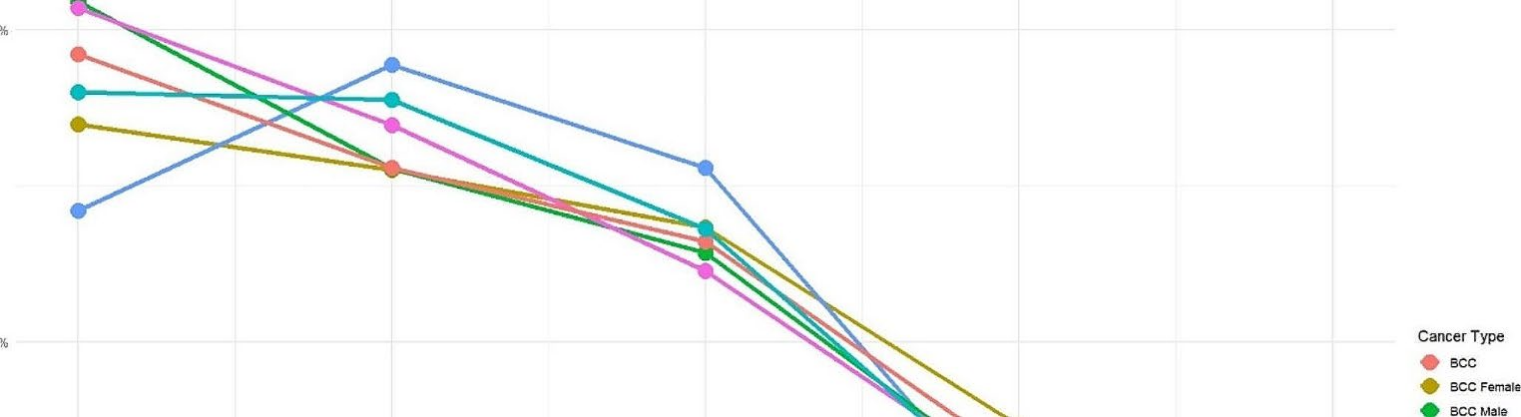
Prevalence of NMSC and NMSC in males and females based on socioeconomic quintiles in New Zealand

The data in Fig. 5 reflects an analysis considering socioeconomic indicators used in New Zealand, with quintile 1 being the least socioeconomically deprived and quintile 5 being the most deprived. The findings show a clear association where the prevalence of NMSC decreases gradually as the economic status of the community declines. Specifically, BCC prevalence drops from an odds ratio (OR) of 3.26(95% CI 3.07–3.45) in the most affluent quintile to 0.65(95% CI 0.56–0.74) in the least affluent quintile. Similarly, the prevalence of SCC reduces from an OR of 2.52(95% CI 2.35–2.68) to 0.52(95% CI 0.45–0.60) across the economic quintiles. For a more detailed overview of NMSC prevalence across genders and quintiles, Fig. 4 and Fig. 5 provide additional information. s Interestingly, male NMSC shows a consistent decrease in prevalence from quintile 1 to quintile 5. However, the prevalence of female SCC appears to decrease linearly from quintile 2 to quintile 5. It must be noted that quintile data is sourced from the government records and were not available for all participants.

## Discussion

Non-melanoma skin cancer (NMSC) is a significant health risk globally, including in New Zealand, but critical gaps in knowledge of the true prevalence exist due to the absence of data collection by the New Zealand Cancer Registry, and many international registries only record melanoma skin cancers. To address this, a comprehensive study on prevalence and epidemiological trends of NMSC in New Zealand was conducted between 2008 and 2022. Because the study was on willing volunteer participants, there may have been an unintended bias towards those more prone to seeking medical attention. However, this large study still provides significant data about NMSC within the New Zealand context and provides valuable insights for other population research. While melanomas were also recorded, this data is captured by cancer registries and is therefore not reported here. Details on the characteristics of patients with multiple melanomas and multiple NMSC will be the basis of further papers.

The main goal of this study was to examine how common non-melanoma skin cancer is in New Zealand, considering age, gender, socioeconomic status, and types of tumours found in patients. Our findings show that the prevalence of basal cell carcinoma (BCC) tends to increase with age, especially after 30 years old, and then the rate stabilizes after 50. Compared to squamous cell carcinoma (SCC), BCC is three times more prevalent in the age group of 31–50 years. On the other hand, SCC becomes more common after age 30 and starts approaching BCC prevalence after age 50. After age 80, SCC is significantly more prevalent than BCC. Overall, we found that the prevalence of BCC has not significantly changed from 2008 to 2022, but we have observed a significant increase in the prevalence of SCC as a percentage, within the study population of fair-skinned New Zealanders. This trend may be due to the ageing population, which could lead to a stabilization of BCC and an increase in SCC but could also be related to climate change. It has been estimated by others that a 2 °C (3.6 °F) increase in ambient temperature could potentially increase skin cancer incidence 11% by 2050 [17].

According to our study, males are more likely to develop NMSC than females. This finding can be explained by various factors, including the higher levels of occupational sun exposure experienced by men in outdoor occupations. Additionally, males possibly tend to have lower adherence to sun protection practices than females, contributing to the higher incidence of NMSC among men. However, it is essential to note that the total number of patients diagnosed with BCC was 3,355 in males, compared to 2,422 for SCC within the study population of over 32,839. Similarly, the total number of BCC cases in females was 2,300 compared to 2,078 for SCC. These figures indicate that males have a higher incidence of BCC than females. Specifically, BCC’s prevalence in males is 1.5 times greater than in females, while SCC in males is only 1.2 times higher than in females.

It has been reported previously that of NMSC subtypes, BCC is the most prevalent, followed by SCC [18]. It is worth highlighting that NMSC is closely associated with cumulative sun exposure over an individual’s lifetime, often resulting from both prolonged and repeated intermittent exposure to sunlight. SCC in-situ are the most frequently observed subtype within SCC.

The second objective of this analysis was to investigate the variations in tumour types across different body anatomical regions. The study of various body sites revealed variations in the prevalence of BCC and SCC, confirming that prolonged exposure to UV light is a significant risk factor for SCC [19] that typically manifests in sun-exposed areas. Our study revealed a higher likelihood of SCC occurrence in the face and limbs compared to the back. Conversely, for BCC, intermittent sun exposure (such as recreational exposure) assumes greater importance as a risk factor more than sustained sun exposure [20]. Our findings also correlated this by indicating a higher probability of BCC occurrence in the face and back than in the limbs. Specifically, BCC prevalence was approximately 2 to 3 times higher than SCC for the back, eyes, neck, and nose. On the other hand, for the lower limbs, upper limbs, and scalp, the prevalence of SCC was found to be 2 to 3 times higher than that of BCC. Similar prevalence of SCC and BCC were observed in the ear, lips, and face.

One major interest was the unexpected finding that female patients have a higher incidence of BCC on their lips, while SCC is more common in the nose and extremities. These differences may be due to factors such as the use of cosmetics such as lipstick on the lips by women.

A previous study [21] had noted a positive correlation between the prevalence of NMSC and the region’s socioeconomic status, which was also our finding. A few factors may explain this connection. For example, as communities experience economic growth, people tend to have more leisure time for outdoor activities that involve sun exposure, like beach activities, outdoor sports, or gardening. Regions with higher economic levels may have an ageing population due to greater longevity, and income may correlate with fairer skin in New Zealand, further contributing to the observed association.

## Conclusion

This study represents the first ever comprehensive assessment of NMSC prevalence in New Zealand that considers across various demographic factors, including age group, gender, economic status, and tumour subtype.

Skin cancer rates are rising, and older ages and higher incomes have an association with higher rates. Overall males have more NMSC than females. BCC is three times more prevalent in the 31-50 age group, whereas SCC are significantly more prevalent after age 80. Prevalence of BCC has not changed over the 15-year timeline of this study but SCC rates have increased.

These findings from New Zealand provide a scientific basis for planning skin cancer screening and intervention strategies. This information in the public domain may also help encourage preventative sun protection measures.

## Data Availability

No datasets were generated or analysed during the current study.
